# Deletion of Individual Ku Subunits in Mice Causes an NHEJ-Independent Phenotype Potentially by Altering Apurinic/Apyrimidinic Site Repair

**DOI:** 10.1371/journal.pone.0086358

**Published:** 2014-01-23

**Authors:** Yong Jun Choi, Han Li, Mi Young Son, Xiao-hong Wang, Jamie L. Fornsaglio, Robert W. Sobol, Moonsook Lee, Jan Vijg, Sandra Imholz, Martijn E. T. Dollé, Harry van Steeg, Erwin Reiling, Paul Hasty

**Affiliations:** 1 Department of Molecular Medicine, The University of Texas Health Science Center at San Antonio, San Antonio, Texas, United States of America; 2 Department of Pharmacology and Chemical Biology, University of Pittsburgh School of Medicine, Pittsburgh, Pennsylvania, United States of America; 3 Hillman Cancer Center, University of Pittsburgh Cancer Institute, Pittsburgh, Pennsylvania, United States of America; 4 Department of Human Genetics, University of Pittsburgh Graduate School of Public Health, Pittsburgh, Pennsylvania, United States of America; 5 Department of Genetics, Albert Einstein College of Medicine, Bronx, New York, United States of America; 6 Center for Health Protection, National Institute for Public Health and the Environment (RIVM), Bilthoven, The Netherlands; 7 MGC Department of Cell Biology and Genetics, Center for Biomedical Genetics, Erasmus MC, Rotterdam, The Netherlands; University of North Dakota, United States of America

## Abstract

Ku70 and Ku80 form a heterodimer called Ku that forms a holoenzyme with DNA dependent-protein kinase catalytic subunit (DNA-PK_CS_) to repair DNA double strand breaks (DSBs) through the nonhomologous end joining (NHEJ) pathway. As expected mutating these genes in mice caused a similar DSB repair-defective phenotype. However, *ku70^-/-^* cells and *ku80^-/-^* cells also appeared to have a defect in base excision repair (BER). BER corrects base lesions, apurinic/apyrimidinic (AP) sites and single stand breaks (SSBs) utilizing a variety of proteins including glycosylases, AP endonuclease 1 (APE1) and DNA Polymerase β (Pol β). In addition, deleting Ku70 was not equivalent to deleting Ku80 in cells and mice. Therefore, we hypothesized that free Ku70 (not bound to Ku80) and/or free Ku80 (not bound to Ku70) possessed activity that influenced BER. To further test this hypothesis we performed two general sets of experiments. The first set showed that deleting either Ku70 or Ku80 caused an NHEJ-independent defect. We found *ku80^-/-^* mice had a shorter life span than *dna-pkcs^-/-^* mice demonstrating a phenotype that was greater than deleting the holoenzyme. We also found Ku70-deletion induced a p53 response that reduced the level of small mutations in the brain suggesting defective BER. We further confirmed that Ku80-deletion impaired BER via a mechanism that was not epistatic to Pol β. The second set of experiments showed that free Ku70 and free Ku80 could influence BER. We observed that deletion of either Ku70 or Ku80, but not both, increased sensitivity of cells to CRT0044876 (CRT), an agent that interferes with APE1. In addition, free Ku70 and free Ku80 bound to AP sites and in the case of Ku70 inhibited APE1 activity. These observations support a novel role for free Ku70 and free Ku80 in altering BER.

## Introduction

BER corrects a broad spectrum of DNA lesions [1] caused by reactive oxygen species (ROS) and alkylating agents [2] that would otherwise result in point mutations [3]. The damaged nucleotide is first recognized by one of many DNA damage specific glycosylases [4]. For example 8-oxoguanosine-glycosylase 1 (OGG1) is the primary glycosylase to excise the major ROS-induced base lesion, 8-oxoG. Glycosylases remove the damaged base to generate an apurinic/apyrimidinic (AP) site. AP endonuclease 1 (APE1) then makes a nick 5′ to the AP site, generating a dRP (deoxyribose phosphate) intermediate and a one base gap. DNA Polymerase β (Pol β) then fills in the missing nucleotide while its lyase activity generates a 5′ phosphorylated DNA strand by excising the 5′ terminal dRP residue so that DNA ligase can repair the nick. BER also repairs DNA single strand breaks (SSBs) that form spontaneously at AP sites, as a DNA repair intermediate or after exposure to ROS. XRCC1 is critical for repairing SSBs by interacting with a number of BER proteins including APE1 [5], [6]–[8], and PARP-1[6], [9]. Thus, deletion of BER components disables the repair of base lesions, AP sites and SSBs[10].

By contrast NHEJ repairs DNA DSBs. To initiate NHEJ, Ku70 and Ku80 form a heterodimer called Ku that forms a holoenzyme with DNA-PK_CS_ [11]. Cells deleted for any of these proteins exhibited telomere end fusion [12], hypersensitivity to clastogenic agents, and premature replicative senescence [13]. Mice deleted for these proteins exhibited premature aging [14]–[18]. Thus, deletion of Ku70, Ku80 or DNA-PK_CS_ resulted in a similar phenotype demonstrating a common defect in the holoenzyme. In addition, XRCC4 and DNA ligase IV form a heterodimer to join the broken ends. Cells deleted for either of these proteins also exhibited hypersensitivity to clastogens, premature replicative senescence [19] and early aging [20]. Thus, deletion of NHEJ proteins caused a similar phenotype.

However, our data also show that cells deleted for either Ku70 or Ku80 exhibited an NHEJ-independent phenotype. We found cells deleted for Ku70 or Ku80 were hypersensitive to ROS and alkylating agents implicating defective BER [21], [22]. However, cells deleted for Lig4 did not exhibit these hypersensitivities exonerating defective NHEJ. In addition, extracts from cells deleted for Ku80, but not Lig4, exhibited reduced BER capacity (correction of a U/G mismatch). Furthermore, ectopic expression of OGG1 or PARP-1 in Ku80-deleted cells rescued hypersensitivity to ROS [22] suggesting Ku80 deletion disabled BER. These data suggested Ku80-deletion caused a BER defect that was unrelated to NHEJ.

We hypothesized that free Ku70 (not bound to Ku80) and free Ku80 (not bound to Ku70) could influence BER. Cells deleted for either Ku70 or Ku80 did not exhibit the same sensitivity to ROS agents [21] and extracts from cells deleted for Ku80, but not Ku70, exhibited reduced BER capacity (correction of a U/G mismatch) [22]. We further hypothesized that free Ku70 could have activity in *ku80^-/-^* cells and free Ku80 could have activity in *ku70^-/-^* cells [21], [22]. This is possible since some Ku80 remains in the absence of Ku70 [23] and vice versa [24]. To support this hypothesis, we found deleting Ku70 resulted in a milder phenotype than deleting Ku80 in p53-mutant mice and this milder phenotype depended on Ku80 [21]. Thus, after deleting Ku70 in p53-mutant mice, there were two events that contributed to the phenotype: first, the loss of Ku70 and second, the activity of free Ku80. It is also possible that free Ku70 could contribute to the Ku80-mutant phenotype.

Here we test our model that proposes free Ku70 and/or free Ku80 alter BER. First, we provide more data that show deletion of either Ku70 or Ku80 caused a phenotype that was not due to defective NHEJ. We found that *ku80^-/-^* mice had a shorter life span than *dna-pkcs^-/-^* mice suggesting a phenotype greater than simply deleting NHEJ. Next we show that *ku70^-/-^* mice exhibited an elevated level of point mutations in the brain but only in the absence of p53 suggesting a defect in BER. We also show that Ku80-deletion in cells inhibited BER through a novel mechanism that is not epistatic to Pol β. Second, we present data that show free Ku70 and free Ku80 influence BER. Deletion of either Ku70 or Ku80, but not both, disabled the repair of AP sites. Interestingly, free Ku70 and free Ku80, but not the Ku heterodimer, associated with AP sites and free Ku70 inhibited APE1 activity *in vitro*. Thus, Ku70 and Ku80 appear to have activity outside NHEJ and the Ku heterodimer that influences BER.

## Results

### Ku80-deleted mice have a shorter life span than DNA-PK_CS_-deleted mice

**Figure 1:**
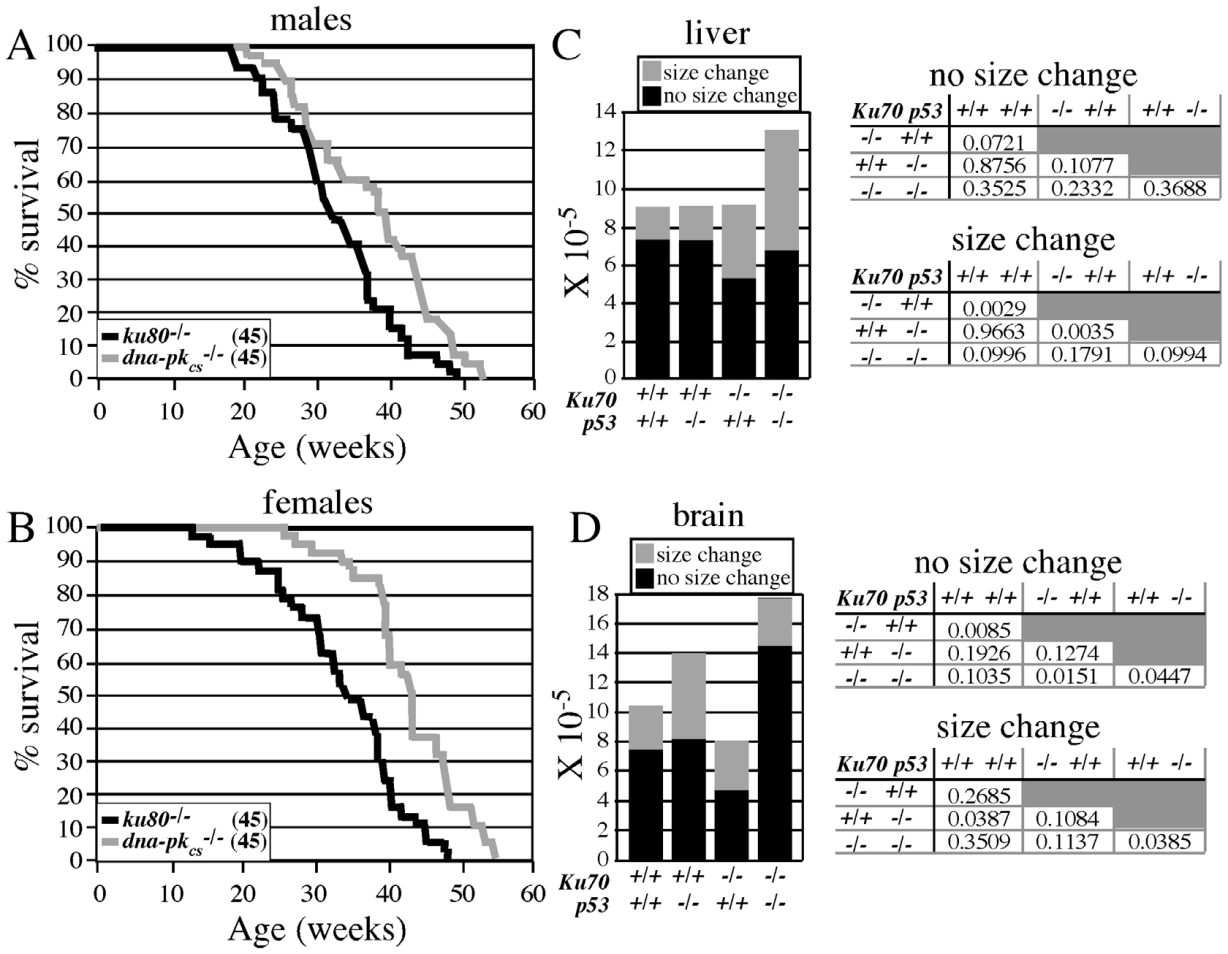
*In vivo* analysis. Life span for *ku80^-/-^* and *dna-pk_cs_^-/-^* (A) males and (B) females (45 mice in each cohort). Mutation spectrum in *ku70^-/-^* mice with and without p53 in the (C) liver and (D) brain. Size changes are chromosomal rearrangements that include translocations and large insertions/deletions. No size changes are point mutations (base changes and small insertions/deletions). A student t test was performed for a statistical analysis and tables are presented showing all possible comparisons.

Deletion of either Ku70 of Ku80 caused any early aging phenotype implicating a defect in NHEJ [16]. Yet in a p53-mutant background the *ku70^-/-^* mice lived significantly longer than the *ku80^-/-^* mice due to a lower incidence of pro-B cell lymphoma [21]. The presence of Ku80 was essential for this milder phenotype. Thus, we proposed that Ku80 functioned outside of the Ku heterodimer. To investigate the possibility that Ku80 functioned outside of the DNA-PK holoenzyme, we measured the life spans for mice deleted for either Ku80 or DNA-PK_CS_ [11]. To guard against phenotypic variation due to differences in genetic background and environment, C57Bl6/J males were crossed to FVB females (both are *Ku80^+/-^ DNA-PK_CS_^+/-^*) such that all mice were C57Bl6/J*FVB F1 hybrid brothers and sisters raised in the same cages. The *ku80^-/-^* mice exhibited a shorter life span than the *dna-pk_cs_^-/-^* mice for both males (Fig. 1A, p = 0.01) and females (Fig. 1B, p<0.0001). Thus, deleting Ku80 is more severe than deleting DNA-PK_CS_. Even though the mechanism for these different life spans is not known, this observation demonstrates that Ku80 has a function greater than the DNA-PK holoenzyme.

### Ku70-mutant mice exhibited an elevated level of point mutations in the absence of p53

Previously we analyzed *ku80^-/-^* mice for the level of chromosomal rearrangements and point mutations in the liver and brain. We found Ku80-deletion increased levels of chromosomal rearrangements in a variety of tissues including the liver, as expected for defective NHEJ [25]. However, there was no significant increase in rearrangements in the brain, instead there was a decrease in point mutations. We surmised that p53-mediated DNA damage responses limited the number of recovered mutations. Here we tested if p53-dependent DNA damage responses influenced the level of mutations in *ku70^-/-^* livers (Fig. 1C) and brains (Fig. 1D) with and without p53 using a forward mutation detection system based on *lacZ*. We crossed the Ku70 mutation [26] and the p53 mutation [27] into mice that carry the pUR288-*lacZ* reporter [28]. These mice were sacrificed at 4 months and analyzed for the levels of rearrangements (size change) and point mutations (no size change) in their livers and brains.

**Figure 2:**
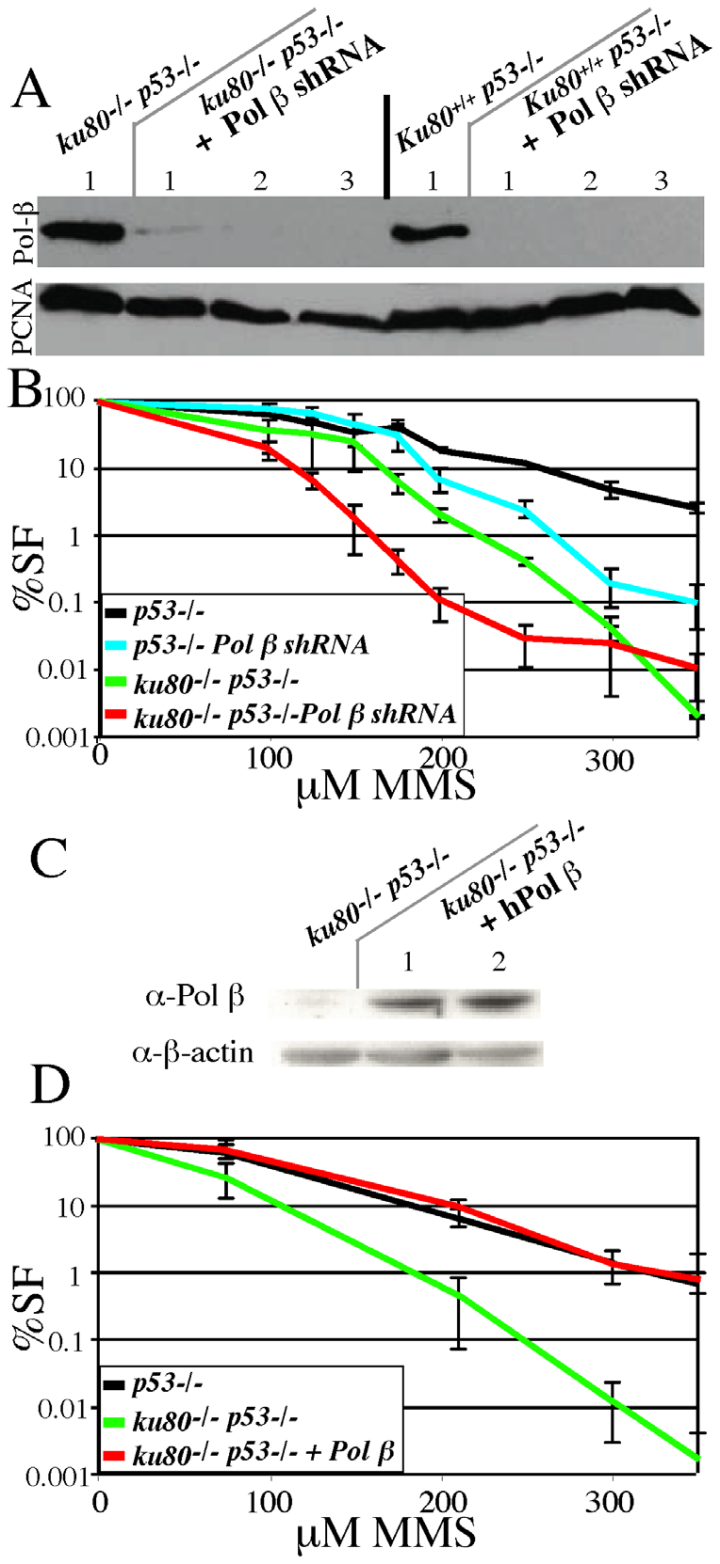
Epistatic analysis for Ku80 and Pol β. All cells are deleted for p53 (even controls) to avoid early replicative senescence. Shown is the average of three experiments. (A) Western showing RNAi knockdown of Pol β in *p53^-/-^* control and *ku80^-/-^ p53^-/-^* fibroblasts that stably express a shRNA plasmid specific for mouse Pol β (three clones). PCNA was loaded to normalize for nuclear protein levels. The expression of endogenous Pol β in the shRNA-transduced cell lines was undetectable by immunoblot, consistent with our earlier reports for this mouse Pol β -specific shRNA [30]. (B) Dose-response to MMS for *p53^-/-^* control and *ku80^-/-^ p53^-/-^* fibroblasts with and without mouse Pol β shRNA expression. (C) Western showing increased Pol β levels for the *ku80^-/-^ p53^-/-^* fibroblasts that stably express a Pol β expression plasmid (two clones). Beta-actin was loaded to normalize for cellular protein levels. (D) Pol β -overexpression rescues Ku80-mutant phenotype for MMS.

First we looked at the level of chromosomal rearrangements. Similar to the results with *ku80^-/-^* mice [25], Ku70-deletion increased chromosomal rearrangements in liver, but not brain. In addition, we found that p53-deletion further increased the levels of chromosomal rearrangements in the liver, but not brain. However, this was not statistically significant because of one outlier among the replicate determinations in the double knockouts (refer to Fig. 2C, D for p values). Overall, however, it is possible to conclude that the liver is more prone to carry chromosomal rearrangements than the brain.

Next we looked at point mutations. Similar to *ku80^-/-^* brains [29], we found decreased levels of small mutations in *ku70^-/-^ p53^+/+^* brains compared to *Ku70^+/+^ p53^+/+^* brains. The levels of small mutations were also lower as compared to *Ku70^+/+^ p53^-/-^* brains, but this was not significant. Yet, *ku70^-/-^ p53^-/-^* brains exhibited increased point mutations as compared to *ku70^-/-^ p53^+/+^* and *Ku70^+/+^ p53^-/-^* brains. Thus, p53 reduced the level of small mutations in the *ku70^-/-^* brain. This observation supports the hypothesis that deletion of either Ku70 or Ku80 increased base lesions that are then limited through p53-mediated responses and corroborate our previous observations with tissue culture cells that either Ku70 and/or Ku80 influence the repair of base lesions [22].

### The increased sensitivity to an alkylating agent in cells compromised for Ku80 and Pol β was additive

The above experiments support the possibility that deleting either Ku70 or Ku80 altered BER in mice. Therefore, we performed an epistatic analysis for Ku80 and Pol β to determine if they repaired methyl methanesulfonate (MMS)-induced lesions via the same sub-pathway [2]. We analyzed mouse embryonic fibroblasts deleted for p53 since NHEJ-deletion caused p53-mediated replicative senescence that prevents their proliferation; therefore, all cells were deleted for p53, including control cells [13], [21]. Pol β was depleted in *p53^-/-^* control and *ku80^-/-^* p53-/- MEFs by RNA interference using a mouse Pol β -specific shRNA-expressing lentivirus similar to one we previously reported [30]. We used this lentivirus to transduce *p53^-/-^* control and *ku80^-/-^ p53^-/-^* MEF to generate corresponding cells with a deficiency in the expression of Pol β (Fig. 2A). Each group of cells was also transduced with a control, GFP-expressing lentivirus. These cells were then used to perform a dose-response analysis to MMS. We found Pol β depletion increased the sensitivity to MMS for control and *ku80^-/-^ p53^-/-^* cells; yet the latter appear more sensitive than the former genotype (Fig. 2B). Thus, Pol β -mediated repair of MMS-induced lesions in both control and *ku80^-/-^ p53^-/-^* MEFs. We also found Pol β over-expression (Fig. 2C) rescued sensitivity to MMS for *ku80^-/-^ p53^-/-^* cells (Fig. 2D). Thus, these data suggest that Pol β and Ku80 are not epistatic and that Ku80 deletion negatively impacts the gap tailoring or DNA synthesis/ligation stages of BER without disabling Pol β [2].

### Free Ku70 and free Ku80 sensitize cells to an APE1 inhibitor

**Figure 3:**
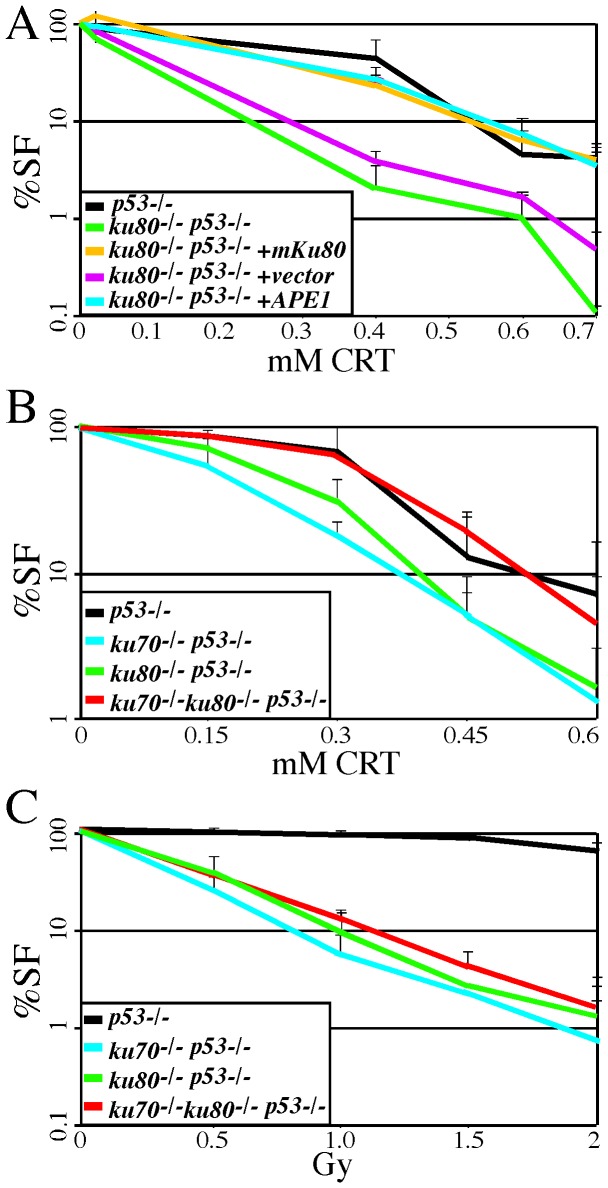
CRT0044876 (CRT) survival fraction (SF). All cells are deleted for p53 (even controls) to avoid early replicative senescence. Shown is the average of three experiments. (A) Cells deleted for Ku80 are hypersensitive to CRT. Expression of APE1 or mouse Ku80 rescued CRT hypersensitivity for Ku80-mutant cells. (B) Cells deleted for either Ku70 or Ku80 but not both were hypersensitive to CRT demonstrating independent function for the individual Ku subunits as opposed to the Ku heterodimer. (C) Cells deleted for Ku70 or Ku80 or both were hypersensitive to γ-radiation demonstrating the Ku heterodimer repaired damage as opposed to independent function for the individual proteins.

Our previously published results in mice [21] and tissue culture cells [22] suggest that either Ku70 or Ku80 function outside of the Ku heterodimer to influence BER. We also found that Ku80-deletion decreased the capacity to repair AP sites [22]. Therefore, we tested APE1 capacity in mouse fibroblasts deleted for Ku70 or Ku80 or both to provide a complementary biological analysis. We found that *ku80^-/-^ p53^-/-^* mouse embryonic fibroblasts [13] were hypersensitive to CRT0044876 (CRT), an agent that specifically inhibits APE1 nicking [31] (Fig. 3A). Expression of mouse Ku80, but not vector alone, rescued hypersensitivity. Ectopic APE1 overexpression also rescued CRT hypersensitivity, supporting the possibility that free Ku70 competitively inhibited APE1 nicking. We then compared *ku70^-/-^ p53^-/-^* and *ku80^-/-^ p53^-/-^* dermal fibroblasts [21] to *ku70^-/-^ ku80^-/-^ p53^-/-^* dermal fibroblasts to determine if free Ku70 caused CRT hypersensitivity in *ku80^-/-^ p53^-/-^* cells and if free Ku80 caused CRT hypersensitivity in *ku70^-/-^ p53^-/-^* cells. We found *ku70^-/-^ p53^-/-^* cells and *ku80^-/-^ p53^-/-^* cells, but not *ku70^-/-^ ku80^-/-^ p53^-/-^* cells, were hypersensitive to CRT (Fig. 3B). Therefore, free Ku70 and free Ku80 were needed for CRT hypersensitivity. By comparison, all three genotypes were similarly hypersensitive to γ-radiation (Fig. 3C) showing the Ku heterodimer was needed to repair the γ-radiation-induced DSBs. Thus, free Ku70 and free Ku80 affected BER, but not NHEJ.

### The individual Ku70 and Ku80 subunits, but not the Ku heterodimer, preferentially bound to a substrate with an AP site

**Figure 4:**
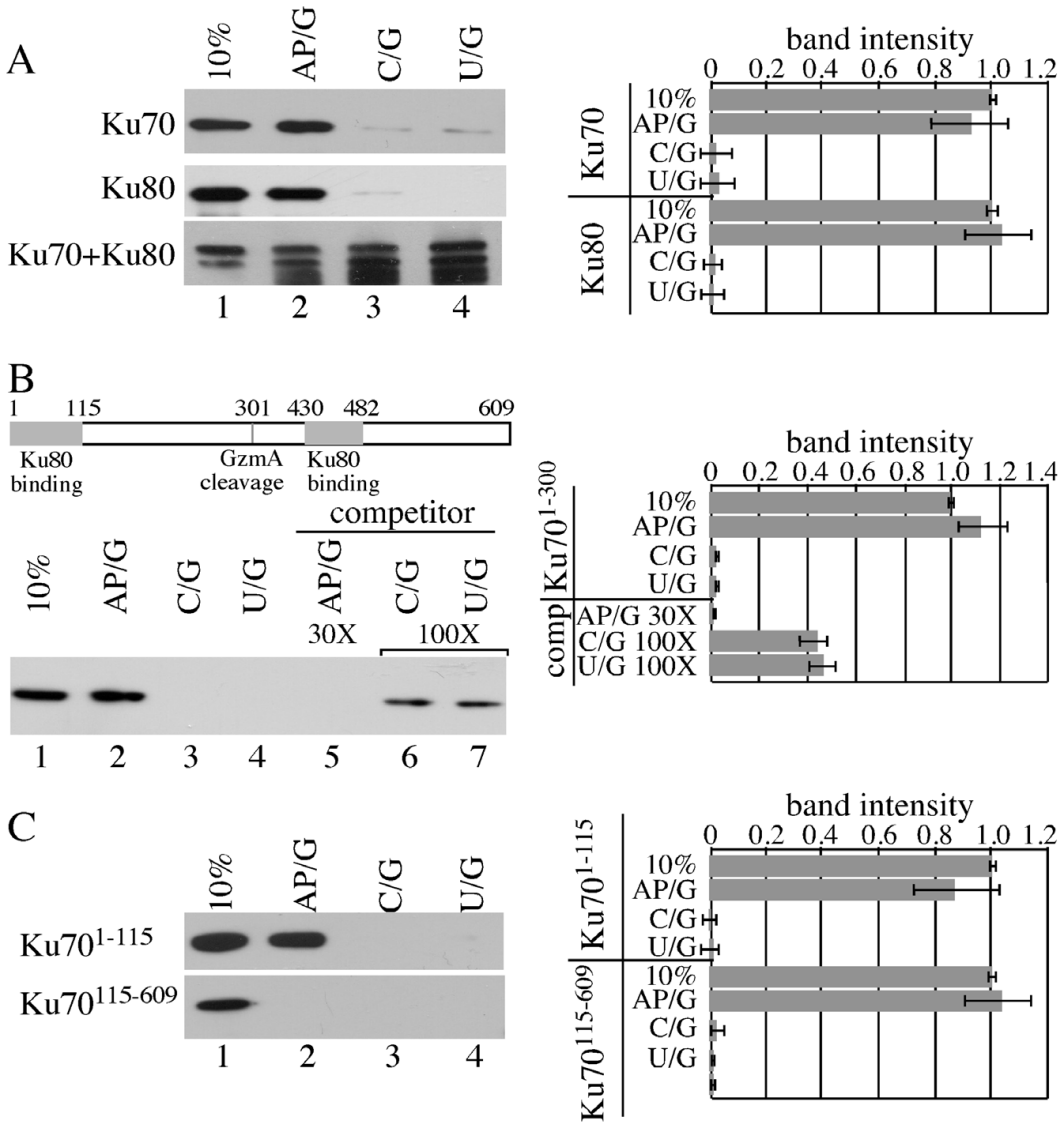
Ku70 and Ku80 bind to AP sites. We show 10% input for *in vitro* translated product. The same concentration of biotinylated substrate were used for each reaction of the binding assays. Right panel shows the relative band intensity as measured with the Kodak document program and as compared to 10% input. Shown is the average of three experiments with error bars (standard deviation). Statistics are shown in the results. (A) Ku70 and Ku80, but not Ku70+Ku80, preferentially bind to the AP site. (B) GzmA cleaves Ku70 at Arg(301). Cleavage at this site separates the two Ku80 binding domains. The GzmA N-terminal Ku70 cleavage product (Ku70^1–300^) binding and competition assays. (C) Ku70^1–115^, but not Ku70^115-609^, bound to the AP/G substrate at a higher level than the C/G and U/G substrates.

Previously Ku80 was shown to crosslink with AP sites in HeLa cell extracts and the Ku heterodimer along with DNA-PK_CS_ was shown to inhibit AP site cleavage by APE1 [32]. For efficient end joining Ku was also shown to process AP sites at DNA ends with Ku70's 5′-dRP/AP lyase activity [33]. Here we analyzed the potential mechanism for the increased CRT sensitivity observed in cells deleted for Ku70 or Ku80, but not both. We hypothesized that free Ku70 and free Ku80 bound to AP sites to interfere with their repair. This is possible since some Ku80 remains in the absence of Ku70 [23] and vice versa [24]. For this purpose we used an oligonucleotide with an internal U/G mismatch and then converted the uracil to an AP site with uracil DNA glycosylase (UDG). Binding assays were performed with this AP/G substrate and the parental U/G substrate or the same substrate with a C instead of a U (C/G substrate). Biotinylated substrates were exposed to myc-tagged Ku70 (myc-Ku70) or myc-tagged Ku80 (myc-Ku80) after *in vitro* translation, diluted on ice with NaBH_4_ to trap proteins that form a Schiff base with the deoxyribose of the AP site [33], [34] and isolated with streptavidin-coated magnetic beads. Western blotting was performed with an anti-myc antibody. Myc-Ku70 exhibited preferential binding to the AP/G substrate as compared to the C/G (Fig. 4A, compare lanes 2 & 3, p = 0.000112, student t test) and U/G (Fig. 4A, compare lanes 2 & 4, p = 0.005) substrates. Myc-Ku80 also exhibited preferential binding to the AP/G substrate as compared to the C/G (Fig. 4A, compare lanes 2 & 3, p = 0.0022) and U/G (Fig. 4A, compare lanes 2 & 4, p = 0.0015) substrates. Thus, free Ku70 and free Ku80 bound to AP sites.

The Ku heterodimer binds to DNA ends [35]; therefore, we tested if Ku would preferentially bind to an oligonucleotide substrate with an AP site by combining full length myc-Ku70 and myc-Ku80. However, unlike myc-Ku70 and myc-Ku80 alone, the combination did not preferentially bind to the AP/C substrate as compared to the C/G (Fig. 4A, compare lanes 2 & 3, p = 0.436) and U/G (Fig. 4A, compare lanes 2 & 4, p = 0.344) substrates. Instead, the Ku heterodimer bound to all substrates, implicating end binding. Thus, the Ku heterodimer did not preferentially bind to an AP site. This observation also suggests that free Ku70 and free Ku80 do not efficiently bind to DNA ends as compared to AP sites. Therefore, free Ku70 and free Ku80, but not the Ku heterodimer, bound to AP sites.

### N-terminal Ku70, but not C-terminal Ku70, preferentially bound to a substrate with an AP site

Mechanistically, it is not clear how free Ku70 and free Ku80 could inhibit AP site repair in wild type cells or animals. One possibility is that one subunit is degraded while leaving the other intact. Granzyme A (GzmA) cleaves Ku70 at Arg301 to separate the two Ku80 binding domains (Fig. 4B) thereby preventing formation of the Ku heterodimer [36] as a part of the caspase-independent cell death pathway in killer cell cytotoxic granules [37]. Interestingly, GzmA also cleaves APE1 to induce cell death; thus, Ku70 cleavage could further diminish BER.

Since GzmA cleaves Ku70 at Arg301, we tested myc-Ku70^1–300^ for its binding preference to these substrates. Similar to full length Ku70 (1–609), Myc-Ku70^1–300^ exhibited preferential binding to the AP/G substrate as compared to the C/G (p = 0.0179) and U/G (p = 0.0131) substrates (Fig. 4B, lanes 2–4). In addition, a competition assay with myc-Ku70^1–300^ was performed with hot (biotinylated) and cold (not biotinylated) substrate. We found 30X cold AP/G competed out hot AP/G when compared to no competitor (Fig. 4B compare lanes 2 & 5, p = 0.00037). However, 100X cold C/G (Fig. 4B compare lanes 2 & 6, p = 0.054) and 100X cold U/G (Fig. 4B compare lanes 2 & 7, p = 0.062) did not compete with the AP substrate to the same extent as 30X cold AP/G. Thus, Ku70^1–300^ (the N-terminal GzmA cleavage product) preferentially bound to AP sites. We also found that preferential binding to the AP/G substrate was seen for myc-Ku70^1–115^ as compared to the C/G (p = 0.031) and U/G (p = 0.036) substrates (Fig. 4C). However, myc-Ku70^115–609^ did not display preferential binding to the AP/G as compared to the C/G (p = 0.226) and U/G (p = 0.166) substrates (Fig. 4C). Thus, myc-Ku70^1–115^ was necessary and sufficient to preferentially bind to the AP/G substrate, suggesting the N-terminal Ku70 cleavage product associates with AP sites to inhibit APE1.

### Full length and N-terminal Ku70, but not C-terminal Ku70 or Ku80, inhibited APE1 activity

**Figure 5:**
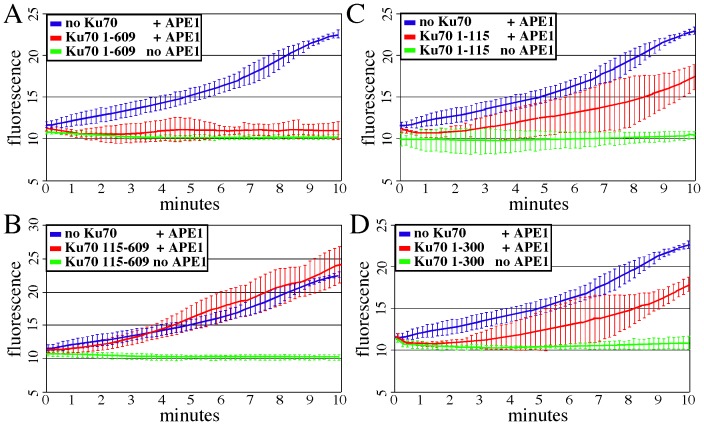
Molecular beacon assay to measure APE1 activity. No Ku70 was compared to Ku70 added to substrate with or without APE1. Fluorescence: the excitation wavelength is 485 nm and the emission wavelength is 538 nm. Shown is the average of three experiments with error bars (standard deviation). (A) Ku70^1–609^ (full-length Ku70) (B) Ku70^115–609^. (C) Ku70^1–115^. (D) Ku70^1–300^.

Free Ku70 and free Ku80 binding to AP sites suggest a possible mechanism for interfering with APE1 activity. This activity was tested using a real-time molecular beacon assay [38] that measures fluorescence emitted after purified APE1 nicks 5′ to the AP site to release a FAM fluorophore from the dabsyl quench located on the complementary DNA strand. APE1 effectively released the FAM fluorophore from the DNA substrate without Ku70 (Fig. 5A, p<0.0001, student t test). Addition of myc-Ku70^1–609^ (full length) prohibited APE1 nicking (Fig. 5A, p<0.0001). However, addition of myc-Ku70^115–609^ did not affect APE1 nicking (Fig. 5B, p = 0.7164). Furthermore, addition of myc-Ku70^1–115^ (Fig. 5C, p<0.0001) myc-Ku70^1–300^ (Fig. 5D, p<0.0001) impaired APE1 as compared to no myc-Ku70, though not as efficiently as full-length myc-Ku70. These data indicate that myc-Ku70^1–115^, myc-Ku70^1–300^ and myc-Ku70^1–609^, but not myc-Ku70^115–609^, inhibited APE1 activity in keeping with the AP binding data. Thus, Ku70 binding to AP sites inhibits APE1 nicking. Interestingly, Ku80 did not inhibit APE1 activity by this assay (not shown), though a previous report showed that Ku and DNA-PK_CS_ inhibited AP site cleavage by APE1 [32].

## Disscussion

Mice and cells deleted for either Ku70 or Ku80 exhibit a complex phenotype that is not solely due to disruption of NHEJ; it is possible some of this complexity is due to altered BER. In mice Ku80-deletion had a more severe impact on life span than DNA-PK_CS_-deletion suggesting Ku80 does more than NHEJ. The level of small mutations also changed in mouse brains deleted for either Ku70 or Ku80 [25] implicating a change in BER. To further implicate altered BER, cells deleted for either Ku70 or Ku80, but not Lig4, exhibited increased sensitivity to ROS and alkylating agents and cells deleted for Ku80, but not Lig4, were deficient in repairing AP sites and cell extracts were deficient in correcting a U/G mismatch in an oligonucleotide substrate [21], [22]. Furthermore, ectopic expression of either OGG1 or PARP-1 recused hypersensitivity of *ku80^-/-^* cells to ROS while ectopic expression of Pol β or APE1 rescued hypersensitivity to an alkylating agent and an APE1 inhibitor, respectively. These rescue experiments suggest that deleting either Ku70 or Ku80 decreased BER capacity but still left the BER pathway intact.

Previously published data support the possibility that Ku70 and Ku80 have activity outside the heterodimer. For example, Ku70 and Ku80 passed through the nucleus using different nuclear localization signals [39], [40] and γ-radiation increased levels of Ku70 but not Ku80 [41]. Ku70 also associated with DNA [42] and with a variety of proteins independent of Ku80. These protein-protein interactions could influence cell death [43], [44], chromatin metabolism[45]–[47] and DNA repair [48]. Thus, Ku70 and Ku80 might function outside the Ku heterodimer to influence a variety of biological outcomes.

Our data also suggest that free Ku70 and free Ku80 could influence the phenotype of cells and mice deleted for their partner in the Ku heterodimer. In a p53-mutant background, we found free Ku80 ameliorated the severity of Ku70-deletion in mice [21]. We also show that deletion of either Ku70 or Ku80, but not both, increased sensitivity to an APE1 inhibitor. Based on these biological observations we observed extracts from cells deleted for Ku80, but not Ku70 or Lig4, were deficient in repairing an oligonucleotide with a U/G mismatch [22]. Now we show that both free Ku70 and free Ku80, but not the Ku heterodimer, preferentially bound to an oligonucleotide with an AP site and that free Ku70 inhibited APE1 activity. Thus, Ku70 and Ku80 have activity independent of the Ku heterodimer that can influence the phenotype of cells and mice deleted for their partner.

The impact free Ku70 and free Ku80 could have on BER might be physiologically relevant to GzmA-mediated cell death. GzmA is a serine protease that rapidly migrates from the cytosol to the mitochondria to cripple electron transport and increase ROS [2]. To further enhance cell death, GzmA cleaves APE1 to disable BER, the pathway mostly responsible for correcting ROS-induced lesions [37]. GzmA also cleaves Ku70 in cytotoxic T lymphocytes and natural killer cells [36] to generate an N-terminal Ku70^1–300^ that binds to AP sites and impair APE1 activity. Our data suggests that Ku70 cleavage will further diminish BER to enhance cell death.

From a larger perspective our data suggests that nonequimolar ratios of Ku70 and Ku80 could impair BER in a wide range of cells, even those that do not express GzmA (or in cells that inappropriately express GzmA). For example, Ku70, but not Ku80, levels decline in lymphocytes [49]. This could lead to inadequate BER that would ultimately result in elevated DNA damage responses or, in the absence of these responses, to elevated point mutations. Thus, non-equimolar ratios of Ku70 and Ku80 could diminish BER and enhance p53-mediated cell clearance. This could contribute to generalized aging since Ku70 and Ku80 levels vary with age [50].

## Materials and Methods

### Ethics statement

All animal work was approved by the ethics committee of the National Institutes for Public Health and the Environment (RIVM), Antonie van Leeuwenhoeklaan, Bilthoven, The Netherlands, IACUC protocol #:99047x.

### Life span analysis

F1 hybrid animals were generated with a C57Bl6/J*FVB background. *Ku80^+/-^* [51] and *DNA-PK_CS_^-/-^* mice [52] were re-derived and back crossed to C57Bl6/JIco (Charles River, France) and FVB/NHan^TM^Hsd (Harlan, Germany) using a speed congenics approach [53]. *ku80^-/-^* and *dna-pk_cs_^-/-^* cohorts were generated using double heterozygous knock out breeders (*Ku80^+/-^ DNA-PK_CS_^-/-^*). All male breeders were on C57Bl6/J-pUR288 and all female breeders on FVB background. Thus, the *ku80^-/-^* and *dna-pk_cs_^-/-^* cohorts are F1 brothers and sisters raised in the same cages to eliminate phenotypic variances due to genetic background and environment. They were kept on a 12-hour light/12-hour dark cycle at a standard temperature of 20°C. CRM pelleted maintenance diet (Special Diet Services, UK) and water supplied ad libitum. Survival (life span) was analyzed so there was no intervention. For the end of life analysis, moribund mice were sacrificed by exsanguination after sedation with an intramuscular injection of a Ketamine-Rompun mixture. The Criteria for euthanizing a moribund mouse was >15% weight loss within 2 weeks, not responsive to touch, prominent appearance of ribs, spine and hips, hunch body position, matted fur, prolapse of the rectum or uterus, or a visible tumor.

This study was carried out in strict accordance with institutional guidelines and regulations. All animal work was approved by the ethics committee of the National Institutes for Public Health and the Environment (RIVM), Antonie van Leeuwenhoeklaan, Bilthoven, The Netherlands, IACUC protocol #:99047x. These were survival studies; therefore, mice were monitored every day without intervention. Moribund mice were sacrificed with ketamine/xylazine anesthesia followed by cervical dislocation and all efforts were made to minimize suffering and discomfort. Criteria for moribund were >15% weight loss within 2 weeks, not responsive to touch, prominent appearance of ribs, spine and hips, hunch body position, matted fur, or a visible tumor.

### Mutation analysis

The mutation spectrum was performed by crossing mice with a mutation in Ku70 [26] and p53 [27] into mice with the pUR288-*lacZ* reporter (line 60, integration sites on chromosomes 3 and 4) [28], [54]. The pUR288-*lacZ* reporter was bred to homozygosity. The animals were maintained in the UTHSCSA's animal facility. They were kept on a 12-hour light/12-hour dark cycle at a standard temperature of 23°C. Standard lab chow (Harlan Teklad, Madison WI) and water were supplied ad libitum. Mice were sacrificed by CO_2_ inhalation followed by cervical dislocation at 4 months of age. DNA preparation, plasmid rescue and mutation analysis was performed as described [25], [55].

### CRT0044876 survival fraction

Dose response assays were performed as previously described for HeLa cells [56].

### *In vitro* translation

For *In vitro* translation we followed the TNT® Quick Coupled Transcription/Translation System (Promega, WI) in 50 µl. For full length Ku70, Ku70 deletions and Ku80 PCR products were cloned into the pCS2/myc expression vector (Invitrogen, Carlsbad, CA) using Xho1 or EcoR1 and Asc1 sites (underlined) that adds an NH2-terminal c-Myc tag.

Ku70 1–609:

Ku70-XhoI-Top (5′-ACCGCTCGAGTCAGGGTGGAGTCATATTACAAA-3′)

Ku70-AscI-Bottom (5′-AAGGCGCGCCTCAGTCCTGGAAGTGCTTGGTGA-3′)

Ku70 1–115:

Ku70-XhoI-Top: 5′-ACCGCTCGAGTCAGGGTGGAGTCATATTACAAA-3′

Ku70N 1-115 Asc1-Bottom: 5′-AAGGCGCGCCTCGTTTTGCACCTGG-3′

Ku70 1–300:

Ku70-XhoI-Top: 5′-ACCGCTCGAGTCAGGGTGGAGTCATATTACAAA-3′

Ku70N 1–300 Asc1-Bottom: 5′-AAGGCGCGCCCTGTACTTGTATTAAAGGTCC-3′

Ku70-115-609:

Ku70-115-609 Xho1-Top: 5′-AACTCGAGCTCGACCAGTTTAAGGGACAAC-3′

Ku70-AscI-Bottom: 5′-AAGGCGCGCCTCAGTCCTGGAAGTGCTTGGTGA-3′

Ku80:

Ku80-EcoRI-Top (5′-TTGAATTCAGTGCGGTCGGGGAATAAGGCAG-3′)

Ku80-AscI-Bottom (5′-AAGGCGCGCCCTATATCATGTCCAATAAATCGTCCA-3′).

### *In vitro* binding assay

To make the double strand substrate, single strand oligonucleotides were mixed in equimolar amounts (250 nM) as measured by UV spectrometry, incubated at 90°C (5 min) and slowly cooled to 24°C. The AP site was generated in the U/G substrate after treatment with *E. coli* UDG (5 U/pmol DNA, 50 mM Tris-HCl pH 8.0, 50 mM NaCl, 50 mM KCl, and 5 mM MgCl_2_). The substrate was biotinylated.

Sequences:

U strand: 5′-biotin-GCCCTGCAGGTCGA**U**TCTAGAGGATCCCCGGGTAC-3′

C strand: 5′-biotin-GCCCTGCAGGTCGA**C**TCTAGAGGATCCCCGGGTAC-3′

Template strand: 5′-GTACCCGGGGATCCTCTAGA GTCGACCTGCAGGGC-3′.

For NaBH_4_ trapping [34] we used 10 µl reaction mixtures (50 mM Tris-HCl pH 8.0, 50 mM NaCl, 15 mM EDTA, 250 nM biotinylated-AP/G substrate) and *in vitro* translation (40 µg) prepared on ice and incubated at 37°C (15 min). Then reaction mixtures were diluted on ice with NaBH_4_ (final concentration 20 mM) and incubated at 0°C (30 min). 10 µg of streptavidin-coated magnetic beads (Dynabeads M280, Dynal) was added to the mixture on a rotator at 4°C (15 min). Beads (diameter ∼2.8 µm, 300pmol biotin-binding sites/mg and washed twice in 50 mM Tris-HCl pH 7.4 and 0.1% BSA to remove NaN_3_) were applied as a suspension (6-7×10^8^ beads/ml, 2 µg of beads in 20 µl) in phosphate-buffered saline (PBS, 0.1% BSA and 0.02% NaN_3_). After incubation with mixture, beads were washed three times (1 ml PBS), resuspended in 20 µl of 1X SDS (50 mM Tris-HCl pH 6.8, 2% SDS, 10% glycerol, 1% β-mercaptoethanol, 12.5 mM EDTA and 0.02% bromophenol blue) and heated (5 min). Soluble fraction was separated by 10% SDS PAGE. Proteins were immunoblotted with mouse anti-human c-Myc antibody (BD Biosciences, MD) according to manufacturer's instructions.

### Competition assay

AP/G competitor has same sequences to AP/G substrate but without 5′ end biotin labeling. The AP site was generated in U/G competitor after treatment with *E. coli* uracil DNA glycosylase (5 U/pmol DNA, 50 mM Tris-HCl pH 8.0, 50 mM NaCl, 50 mM KCl, and 5 mM MgCl_2_). C/G competitor has same sequences to C/G substrate without 5′ end biotin labeling. U/G competitor has same sequences to U/G substrate without 5′ end biotin labeling. For competition assay, each tube contained AP/G substrate (50 nM) with AP/G competitor (1.5 µM), C/G competitor (5 µM) and U/G competitor (5 µM) and did same process the incubation with in vitro translation samples, after that pulled down with streptoavidin beads and trapped by treating NaBH_4_. After washing, the beads were resuspended in 20 µl of 1X SDS sample buffer and heated for 5 min and soluble fraction were separated by 10% SDS PAGE. Proteins were immunoblotted with mouse anti-human c-Myc antibody (BD Biosciences, MD) according to manufacturer's instructions.

### Molecular beacon assay for APE1 activity

APE1 activity was measured using a modification of our recently described BER molecular assay [57]–[59]. All oligonucleotides were purchased from Sigma-Aldrich (Saint Louis, MO): Top-FAM-5′-GAGAAΦATAGTCGCC-3′ Bottom-DAB-3′-CTCTTGTATCAGCGC-5′ [Φ; Tetrahydrofuran (THF)]. Oligonucleotides were mixed in equimolar amount (250 nM), heated at 90°C for 5 min, and then slowly cooled down to room temperature to form a double stranded DNA substrate. An oligonucleotide containing a THF AP site mimic was used in the APE1 molecular beacon substrate. The top strand was labeled on the 5′ end with a FAM fluorophore, the bottom on the 3′end with a dabsyl (DAB) quench. Upon APE1 cleavage, the fluorophore dissociated from the dabsyl quench, causing an increase in fluorescence, essentially as described [57]–[59]. All APE1 (New England Biolabs, Ipswich, MA) cleavage reactions were carried out in a buffer containing 20 mM Tris-acetate, pH 7.9, 10 mM Magnesium acetate, 50 mM Potassium acetate, 1 mM dithiothreitol. All reactions were performed at 37°C. Excitation and emission wavelengths for time curves were 485 and 538 mM, respectively. Standard reaction mixtures (100 µl) for the molecular beacon assay contained 20 mM Tris-acetate, pH 7.9, 10 mM Magnesium acetate, 50 mM Potassium acetate, 1 mM dithiothreitol, 250 nM AP DNA duplex and *in vitro* translation samples (40 µg) and APE1 (10 u) were prepared on ice and then measured at 37°C, every 10 sec. for 10 min.
